# Allergic rhinitis associated with the degree of pulmonary involvement due to COVID-19 in patients from a peruvian general hospital

**DOI:** 10.17843/rpmesp.2023.401.12491

**Published:** 2023-03-30

**Authors:** Bianca García-Gallo, Giancarlo Gonzales-Caldas, Diego Urrunaga-Pastor, Percy Herrera-Añazco

**Affiliations:** 1 Universidad Peruana de Ciencias Aplicadas, Lima, Peru. Universidad Peruana de Ciencias Aplicadas Universidad Peruana de Ciencias Aplicadas Lima Peru; 2 Universidad Científica del Sur, Lima, Peru. Universidad Científica del Sur Universidad Científica del Sur Lima Peru; 3 Peruvian Collective Health Network, Lima, Peru. Peruvian Collective Health Network Lima Peru

**Keywords:** Rhinitis, Allergic, Coronavirus Infections, Lung Injury, Patient Acuity

## Abstract

**Objectives.:**

To evaluate the association between allergic rhinitis and the degree of pulmonary involvement in patients with COVID-19 and to determine the frequencies of the main variables.

**Materials and methods.:**

An observational, cross-sectional and analytical study was carried out by reviewing the medical records of patients diagnosed with COVID-19 from the Cayetano Heredia National Hospital between 2020 and 2021. We obtained information regarding the history of allergic rhinitis; pulmonary involvement was assessed by non-contrast tomography results using the chest computed tomography (CT) score. Data regarding sociodemographic and clinical variables was also obtained. Both crude (PR) and adjusted (aPR) prevalence ratios with their respective 95% confidence intervals (CI) were estimated. We also used a generalized linear Poisson family model with log link function and robust variances.

**Results.:**

We evaluated 434 patients, who were mostly male, older than 60 years and had no relevant medical history. Of these, 56.2% had a history of allergic rhinitis and 43.1% had moderate to severe pulmonary involvement. The adjusted regression model showed that the history of allergic rhinitis reduced the severity of COVID-19 according to the pulmonary involvement assessed by the CT score (aPR: 0.70; 95%CI: 0.56-0.88; p=0.002).

**Conclusions.:**

The history of allergic rhinitis resulted in a 30.0% decrease in COVID-19 severity according to the CT score in hospitalized patients.

## INTRODUCTION

Coronavirus disease (COVID-19), caused by the SARS-CoV-2 virus, is a public health problem with almost 658 million confirmed cases and more than six million deaths by December 2022 ^1^. As a result, different risk factors associated with a worse prognosis of the disease were studied during the pandemic, in an effort to design strategies for early intervention and to avoid hospitalizations and deaths. These factors include being male, advanced age, smoking ^2^, diabetes mellitus ^3^, arterial hypertension (HT), cardiovascular disease, obesity, chronic respiratory disease and cancer ^4^. In addition, allergic rhinitis has been suggested to be a potential predictor of worse clinical outcomes in infected patients, although the evidence is controversial ^5^^,^^6^.

A systematic review reported that patients with COVID-19 and allergic rhinitis are less prone to severe disease and hospitalization ^7^. However, the included studies did not specify whether patients had comorbidities that might influence severity of illness, and definitions of severe illness were heterogeneous ^7^. Although the reasons for this association are not entirely clear, elevated concentrations of eosinophilic cationic protein, IL-17 and IL-33 are found in patients with allergic rhinitis ^8^, the latter being present in patients with severe COVID-19 ^9^, which could partially explain this association. Since allergic rhinitis is a common disease, with an incidence of 10 to 40% of the world population ^10^, its possible role as a risk factor for a worse clinical outcome of COVID-19 should be considered.

Peru is one of the countries with the highest mortality rates due to COVID-19 worldwide. As of March 10, 2023, reports showed 4,487,553 confirmed cases and 219,539 deaths nationwide ^1^. Likewise, in Peru, the prevalence of allergic rhinitis can reach up to 23% in Lima ^11^, above the average in Latin America ^12^, due to factors like air pollution ^13^. In this sense, identifying a possible association with pulmonary involvement in infected patients could be relevant. Although some studies attempted to estimate the role of allergic rhinitis as a possible predictor of adverse outcomes in COVID-19 patients, these did not include relevant control variables and used several outcomes ^7^. The tomographic score (TS) is a surrogate variable for pulmonary involvement in infected patients and its increase is associated with increased mortality and disease severity ^14^. In addition, it predicts hospital stay and need for intubation ^15^^,^^16^, so we consider it a good marker to assess the association. Therefore, this study aimed to estimate the association between allergic rhinitis and the degree of pulmonary involvement by COVID-19 in adults from a Peruvian general hospital.

**KEY MESSAGES**

Motivation for the study. The prevalence of allergic rhinitis is frequent in the Peruvian population and its association with the severity of COVID-19 infection has been previously proposed. However, those studies have not adjusted this association for several comorbidities, besides assessing different outcomes.

Main findings. A history of allergic rhinitis was associated with a decrease in COVID-19 severity according to TS in hospitalized patients.

Implications. History of allergic rhinitis should be evaluated in patients with COVID-19, in order to prioritize care.

## MATERIALS AND METHODS

### Design and study population

This is an observational, analytical and cross-sectional study. We included the medical records of hospitalized patients with COVID-19 from the Cayetano Heredia Hospital during the period 2020-2021.

We assessed the medical records of adult patients (18 to older) diagnosed with COVID-19 by clinical evaluation, molecular or antigen testing, or by computed tomography. The history of allergic rhinitis was defined according to the medical records. Patients whose medical records were illegible or who provided incomplete information on the variables of interest were excluded. Similarly, patients diagnosed with COVID-19 by rapid antibody test were excluded.

**Figure 1:**
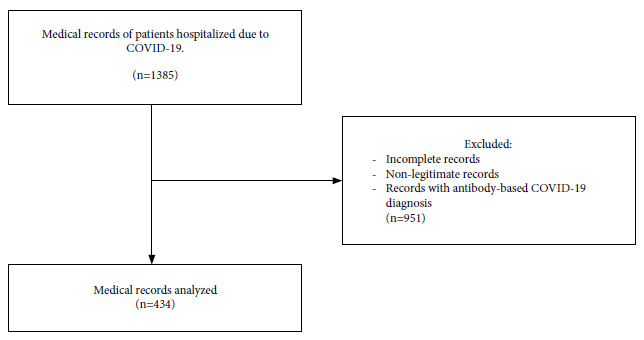
Sample selection flow chart.

Of 434 eligible medical records, we found 245 patients with COVID-19 and complete information regarding the variables of interest; Figure 1 shows the selection flow chart.

### Sample size calculation

The sample was calculated by considering a percentage of unexposed positives of 43% and 57% of exposed positives, with a ratio between both groups of 0.74 ^6^; as well as a confidence level of 95% and a power of 80% were considered. As a result, the calculated sample size was 390 medical records, to which 10% was added on account for lost information, resulting in 434 medical records as the final sample size, which were chosen by non-probabilistic convenience sampling.

### Variables

The independent variable was the history of allergic rhinitis obtained from the patient's self-report during an interview, which was found in the medical records. The dependent variable was the degree of pulmonary involvement due to COVID-19, which was evaluated by means of a non-contrast pulmonary tomography taken after hospital admission and assessed by means of the TS applied by a radiologist. The score ranges from 0 to 25 ^16^, and was categorized into two groups, 0 to 14 and 15 to 25, where the first group included patients with mild involvement and the second group included those with moderate to severe involvement ^15^.

The sociodemographic variables we considered were sex and age. We also included smoking, diabetes *mellitus*, arterial hypertension, cardiovascular disease ^17^, chronic obstructive pulmonary disease (COPD), pulmonary tuberculosis and human immunodeficiency virus (HIV) infection. Data on these conditions were obtained directly from the medical records, as registered by the attending physician. In addition, the history of any neoplasm and the status of the neoplasm, if any, were also considered. Four possible categories were considered to assess the status of the neoplasm: no neoplasm, neoplastic disease in remission, active neoplastic disease under treatment and neoplastic disease without treatment. In addition, we constructed a variable that defined the presence of none, one or two or more comorbidities, considering the previously described conditions (diabetes *mellitus*, HT, cardiovascular disease, COPD, pulmonary tuberculosis and HIV infection). Finally, we considered whether the patient had chronic consumption of oral or inhaled corticosteroids (defined as use for more than 10 days up to admission according to the medical record) ^18^.

### Statistical analysis

Stata 16 statistical program (Stata Corporation, College Station, Texas, USA) was used for the statistical analysis. Categorical variables were described using frequencies and percentages. Likewise, numerical variables were presented with measures of central tendency and dispersion. The proportion between categorical variables were compared with the chi-square test and Fisher's exact test when more than 20% of expected values were less than 5. In addition, the means between two categories of a quantitative variable were compared with the Student's t-test. Finally, a generalized linear Poisson family model with log link function and robust variances was applied to estimate the association between history of allergic rhinitis and pulmonary involvement due to COVID-19. An adjusted model was developed based on epidemiological criteria, considering potential confounders described in the literature. These confounders had to be associated with the exposure variable, outcome and not be found in the causal pathway. In addition, possible collinearity was assessed by a value greater than 10 of the variance inflation factor. Crude prevalence ratios (PR) and adjusted prevalence ratios (aPR) were estimated considering 95% confidence intervals (95%CI).

### Ethical aspects

The original protocol and the survey were approved by the Ethics Committee of the Universidad Peruana de Ciencias Aplicadas on November 22, 2021, in Lima (FCS-SCEI/1152-11-21) and by the Ethics Committee of the Cayetano Heredia Hospital on April 29, 2022, in Lima (OFICIO Nº1231-2022-DG-640-OEGRRHH-360-OADI/HCH). Data for this research was collected from medical records while maintaining the anonymity of the patients, thus making it impossible to identify them. The research team responsible for this project guaranteed respect for the principles of autonomy, justice and non-maleficence. There were no conflicts of interest while conducting this research. The project was registered in the INS-PRISA platform (code: EI00000002619).

## RESULTS

We assessed 434 medical records. The patients were mostly male, over 60 years of age with no significant medical history; 56.2% had a history of allergic rhinitis and 43.1% had moderate to severe pulmonary involvement. Other characteristics are shown in Table 1.

**Table 1:** Descriptive and bivariate analysis of the variables of interest according to allergic rhinitis in patients with COVID-19 from the Cayetano Heredia Hospital in Lima, 2020-2021 (n=434).

Variables	N	%	Allergic rhinitis		p-value
			No	Yes	
			n=190 (43.8%)	n=244 (56.2%)	
Sex					0.695 ^a^
Women	160	36.9	72 (45.0)	88 (55.0)	
Men	274	63.1	118 (43.1)	67 (56.9)	
Age					
Mean ± SD	62.5 ± 13.9		66.7 ± 12.2	59.2 ± 14.3	<0.001 ^c^
18-59	161	37.1	39 (24.2)	122 (75.8)	<0.001 ^a^
60 or more	273	62.9	151 (55.3)	122 (44.7)	
Cancer					0.978 ^b^
Does not have	408	94.0	179 (43.9)	229 (56.1)	
Neoplastic disease in remission	16	3.7	7 (43.7)	9 (56.3)	
Active neoplastic disease under treatment	4	0.9	2 (50.0)	2 (50.0)	
Untreated active neoplastic disease	6	1.4	2(33.3)	4(66.7)	
Smoker					0.764 ^a^
Yes	419	96.5	184 (43.9)	235 (56.1)	
No	15	3.5	6 (40.0)	9 (60.0)	
Comorbidities					0.095 ^a^
0	178	41.0	77 (43.3)	101 (56.7)	
1	88	20.3	47 (53.4)	41 (46.6)	
2 or more	168	38.7	66 (39.3)	102 (60.7)	
Diabetes *mellitus*					0.088 ^a^
No	272	62.7	122 (44.8)	150 (55.2)	
Controlled	54	12.4	29(53.7)	25(46.3)	
Not controlled	108	24.9	39(36.1)	69 (63.9)	
Cardiovascular disease					0.613 ^a^
No	394	90.8	174 (44.2)	220 (55.8)	
Yes	40	9.2	16 (40.0)	24 (60.0)	
Arterial hypertension					0.017 ^a^
No	301	69.4	133 (44.2)	168 (55.8)	
Controlled	87	20.0	45 (51.7)	42 (48.3)	
Not Controlled	46	10.6	12 (26.1)	34 (73.9)	
COPD					0.507 ^b^
No	432	99.5	190 (44.0)	242 (56.0)	
Yes	2	0.5	0 (0.0)	2 (100.0)	
Chronic use of corticosteroids					0.010 ^a^
No	343	79.1	161 (46.9)	182 (53.1)	
Yes	91	20.9	29 (31.9)	62 (68.1)	
Tuberculosis					0.475 ^b^
No	426	98.2	188 (44.1)	238 (55.9)	
Yes	8	1.8	2 (25.0)	6 (75.0)	
HIV					0.507 ^b^
No	432	99.5	190 (43.9)	242 (56.1)	
Yes	2	0.5	0 (0.0)	2 (100.0)	
Pulmonary involvement					0.001 ^a^
Mild (0-14)	247	56.9	91 (36.8)	156 (63.2)	
Moderate/Severe (15-25)	187	43.1	99 (52.9)	88 (47.1)	

SD: standard deviation; COPD: chronic obstructive pulmonary disease; HIV: human immunodeficiency virus.

a Chi-square test, ^b^ Fisher's exact test, ^c^ Student's t-test.

The bivariate analysis showed statistically significant differences between the presence or absence of allergic rhinitis and age groups (p<0.001), history of HT (p=0.017) and chronic consumption of corticoids (p=0.010). Patients with allergic rhinitis had lower mean age (59.2 vs. 66.7), a higher prevalence of uncontrolled HT (73.9% vs. 26.1%), chronic corticosteroid use (68.1% vs. 31.9%) and pulmonary involvement (63.2% vs. 36.8%), compared to those who did not have allergic rhinitis (Table 1).

Statistically significant differences were found during the bivariate analysis between pulmonary involvement and the variables age (p=0.015) and chronic corticosteroid use (p=0.020). Those with moderate to severe pulmonary involvement had a higher mean age (64.4 vs. 61.1) and higher frequency of chronic corticosteroid consumption (53.9% vs. 46.2%) compared to those with mild involvement (Table 2).

**Table 2:** Descriptive and bivariate analysis of the variables of interest according to pulmonary involvement in patients with COVID-19 from the Cayetano Heredia Hospital in Lima in the period 2020-2021 (n=434).

Variables	Pulmonary involvement		
	Mild (0-14)	Moderate/Severe (15-25)	p-value
	n=247 (56.9%)	n=187 (43.1%)	
Rhinitis			0.001 ^a^
No	91 (47.9)	99 (52.1)	
Yes	156 (63.9)	88 (36.1)	
Sex			0.415 ^a^
Women	87 (54.4)	73 (45.6)	
Men	160 (58.4)	114 (41.6)	
Age			
Mean ± SD	61.1 ± 13.5	64.4 ± 14.3	0.015 ^c^
18-59	106 (65.8)	55 (34.2)	0.004 ^a^
60 or more	141 (51.7)	132 (48.3)	
Cancer			0.735 ^b^
Does not have	234 (57.4)	174 (42.6)	
Neoplastic disease in remission	9 (56.3)	7 (43.7)	
Active neoplastic disease under treatment	2 (50.0)	2 (50.0)	
Untreated active neoplastic disease	2 (33.3)	4 (66.7)	
Smoker			0.178 ^a^
Yes	241 (57.5)	178 (42.5)	
No	6 (40.0)	9 (60.0)	
Comorbidities			0.600 ^a^
0	106 (59.6)	72 (40.4)	
1	50 (56.8)	38 (43.2)	
2 or more	91 (54.2)	77 (45.8)	
Diabetes *mellitus*			0.974 ^a^
No	155 (57.0)	117 (43.0)	
Controlled	30 (55.6)	24 (44.4)	
Not controlled	62 (57.4)	46 (42.6)	
Cardiovascular disease			0.053 ^a^
No	230 (58.4)	164 (41.6)	
Yes	17 (42.5)	23 (57.5)	
Arterial hypertension			0.916 ^a^
No	173 (57.5)	128 (42.5)	
Controlled	49 (56.3)	38 (43.7)	
Not Controlled	25 (54.3)	21 (45.7)	
EPOC			0.185 ^b^
No	247 (57.1)	185 (42.9)	
Yes	0 (0.0)	2 (100.0)	
Chronic use of corticosteroids			0.020 ^a^
No	205 (59.8)	138 (40.2)	
Yes	42 (46.2)	49 (53.8)	
Tuberculosis			0.730 ^b^
No	243 (57.1)	183 (42.9)	
Yes	4 (50.0)	4 (50.0)	
HIV			0.185 ^b^
No	247 (57.1)	185 (42.9)	
Yes	0 (0.0)	2 (100.0)	

SD: standard deviation; COPD: chronic obstructive pulmonary disease; HIV: human immunodeficiency virus.

a Chi-square test, ^b^ Fisher's exact test, ^c^ Student's t-test.

**Table 3:** Crude and adjusted prevalence ratio between allergic rhinitis and pulmonary involvement in the Cayetano Heredia hospital in Lima, 2020-2021.

Variable	Crude			Adjusted		
	PR	95%CI	p-value	aPR ^a^	95%CI	p-value
Allergic rhinitis						
No	Reference			Reference		
Yes	0.69	0.56-0.86	<0.001	0.70	0.56-0.88	0.002

PR: crude prevalence ratio; aPR: adjusted prevalence ratio; CI: confidence interval.

a adjusted for: sex, age, being smoker, comorbidities and chronic corticosteroid use.

The crude model analysis showed that a history of allergic rhinitis decreased the severity of COVID-19 (PR: 0.69; 95%CI: 0.56-0.86; p<0.001). This association maintained its direction and magnitude in the regression model adjusted for sex, age, tobacco use, comorbidities and chronic corticosteroid use (aPR: 0.70; 95%CI: 0.56-0.88; p=0.002) (Table3). The complete crude and adjusted regression models are presented in the Supplementary Material.

## DISCUSSION

We found that a history of allergic rhinitis was associated with a 30.0% lower prevalence of moderate to severe pulmonary involvement in patients with COVID-19.

Our findings are similar to those from a systematic review of nine studies, which found that patients with COVID-19 and allergic rhinitis are less prone to severe disease (odds ratio [OR ]: 0.79, 95% CI: 0.52-1.18; p=0.250) and hospitalization (OR: 0.23, 95 %CI: 0.02-2.67; p≤0.001) than patients without allergic rhinitis ^7^. However, the authors point out that the analyzed studies did not assess other comorbidities such as advanced age, diabetes or cardiovascular disease, which, being factors associated with worse outcomes in these patients ^19^, may limit the interpretation of their results. In this sense, our study, by including several comorbidities, provides a more robust analysis of the association. In addition, unlike the studies included in the systematic review, we used TS as a clinical outcome. TS has been described as a useful surrogate predictor of mortality, disease severity ^14^, hospital stay and need for intubation ^15^, therefore, it is a marker that can be used in clinical practice to assess disease severity.

Although the reasons why a history of allergic rhinitis is a protective factor for moderate to severe pulmonary involvement in patients with COVID-19 are unclear, some hypotheses have been proposed. One study found that cat nasal allergen produced a reduction in angiotensin-converting enzyme-2 (ACE2) mRNA expression in the nasal cilia of adult patients with allergic rhinitis who were allergic to cats ^20^. This is relevant, as COVID-19 uses a host cell ACE2 receptor for entry, which can be found on the oral mucosa and the airways ^7^. Furthermore, this receptor plays a notable role in the development of the disease, associated pulmonary lesion and severity of the disease ^21^. Similarly, it has been proposed that exposure to IL-13 would reduce the expression of ACE2 in airway epithelial cells of atopic individuals ^22^. In this sense, it is possible that ACE2 mRNA expression may decrease in cases of allergic rhinitis, which could explain this presumed protection. Additionally, it is possible that the history of rhinitis is not the cause of this protective effect, but rather the drugs used for its treatment, as some studies have suggested. Thus, some research suggests a direct antiviral activity against SARS-CoV-2 virus in H1 receptor antagonists by interfering with the first steps of viral replication or by binding to ACE2 ^23^. Therefore, patients taking these drugs had a significantly lower risk of SARS-CoV-2 infection ^24^. According to previous studies, the prevalence of allergic rhinitis in patients positive to COVID-19 is 57.4% ^6^ which is very similar to our results.

Although our findings are preliminary, they encourage further research on the mechanisms by which a history of allergic rhinitis could be a possible protective factor for adverse clinical outcomes in infected patients. Understanding this potential mechanism could identify therapeutic targets that would allow the development of drugs to treat new infecting variants or other similar viral infections.

The limitations of our study include the use of non-probabilistic sampling and the fact that the results cannot be applied to other populations. Since we used self-reported information, recall bias may have affected our data. Asking about the history of allergic rhinitis is not part of the normal evaluation, so there is a possibility that some physicians may inquire about it and others may not. Relevant variables such as history of asthma, its severity or treatment were not included in the study. In addition, information about the diagnosis of allergic rhinitis did not include the exact time of the disease, severity, treatment or time from the onset of symptoms until the imaging tests. In almost every case, the CT scans may have been taken when pulmonary involvement had not yet reached its maximum extent. No information was collected on time of hospitalization, intensive care unit (ICU) requirement, mechanical ventilation or death. Our study uses pulmonary involvement by TS as a surrogate variable to assess the severity of the clinical picture by COVID-19. Nevertheless, the results suggest a potential factor whose mechanisms should be explored in future research.

In conclusion, more than half of the patients had a history of allergic rhinitis and four out of ten had moderate to severe pulmonary involvement. The history of allergic rhinitis could have a protective effect on moderate to severe pulmonary involvement due to COVID-19. This information will be useful to carry out studies with greater scientific rigor such as cohorts and cases and control studies, in addition to studies that may help explaining the biochemical and molecular mechanism of the protective effect of rhinitis. If the mechanism is found, it could be used for pharmacological purposes.
