# Supplementary material for: Allergic rhinitis associated with the degree of pulmonary involvement due to COVID-19 in patients from a peruvian general hospital
Source: Rev Peru Med Exp Salud Publica. 2023 Mar 30;40(1):51–8. doi: 10.17843/rpmesp.2023.401.12491 (PMC10953649; doi:10.17843/rpmesp.2023.401.12491)
Supplement: Supplementary material. — Available in the electronic version of the RPMESP. [file rpmesp-40-01-12491-s001.docx]

| **Tabla S1.** Modelo de regresión crudo y ajustado para estimar la razón de prevalencia cruda y ajustada entre rinitis alérgica y compromiso pulmonar del hospital Cayetano Heredia en Lima durante el periodo 2020-2021. | | | | | | | | |  |
| --- | --- | --- | --- | --- | --- | --- | --- | --- | --- |
|  | **Crudo** | | |  |  | **Ajustado** | | | |
| **Variables** | **RP** | **IC 95%** | **Valor p** |  |  | **RPa** | **IC 95%** | **Valor p** | |
| Rinitis alérgica |  |  |  |  |  |  |  |  | |
| No | Referencia | - | - |  |  | Referencia | - | - | |
| Sí | 0,69 | 0,56-0,86 | <0,001 |  |  | 0,70 | 0,56-0,88 | 0,002 | |
| Sexo |  |  |  |  |  |  |  |  | |
| Mujer | Referencia | - | - |  |  | Referencia | - | - | |
| Varón | 0,91 | 0,73-1,14 | 0,411 |  |  | 0,92 | 0,74-1,15 | 0,468 | |
| Edad |  |  |  |  |  |  |  |  | |
| 18-59 | Referencia | - | - |  |  | Referencia | - | - | |
| 60 a más | 1,42 | 1,11-1,81 | 0,006 |  |  | 1,23 | 0,95-1,60 | 0,114 | |
| Fumador |  |  |  |  |  |  |  |  | |
| Sí | Referencia | - | - |  |  | Referencia | - | - | |
| No | 1,41 | 0,92-2,17 | 0,114 |  |  | 1,35 | 0,87-2,10 | 0,182 | |
| Comorbilidades |  |  |  |  |  |  |  |  | |
| 0 | Referencia | - | - |  |  | Referencia | - | - | |
| 1 | 1,07 | 0,79-1,44 | 0,668 |  |  | 0,97 | 0,72-1,31 | 0,855 | |
| 2 o más | 1,13 | 0,89-1,44 | 0,313 |  |  | 1,05 | 0,82-1,34 | 0,720 | |
| Consumo crónico de corticoides |  |  |  |  |  |  |  |  | |
| No | Referencia | - | - |  |  | Referencia | - | - | |
| Sí | 1,34 | 1,06-1,68 | 0,013 |  |  | 1,40 | 1,11-1,76 | 0,004 | |
| RP: razón de prevalencia cruda, RPa: razón de prevalencia ajustada; IC: intervalo de confianza. | | | | | | | | |  |

**Material suplementario**
