# Supplementary material for: Allergic rhinitis associated with the degree of pulmonary involvement due to COVID-19 in patients from a peruvian general hospital
Source: Rev Peru Med Exp Salud Publica. 2023 Mar 30;40(1):51–8. doi: 10.17843/rpmesp.2023.401.12491 (PMC10953649; doi:10.17843/rpmesp.2023.401.12491)
Supplement: Translation

# Translation: Rinitis alérgica asociada al grado de compromiso pulmonar por COVID-19 en pacientes de un hospital general peruano

**Authors:** Bianca García-Gallo, Giancarlo Gonzales-Caldas, Diego Urrunaga-Pastor, Percy Herrera-Añazco

**Affiliations:** 1 Universidad Peruana de Ciencias Aplicadas, Lima, Perú.; 2 Universidad Científica del Sur, Lima, Perú.; 3 Red Peruana de Salud Colectiva, Lima, Perú.

**Keywords:** Rinitis Alérgica, Infecciones por Coronavirus, Lesión Pulmonar, Gravedad del Paciente

## Abstract

**Objetivos.:**

Evaluar la asociación entre rinitis alérgica y el grado de compromiso pulmonar en pacientes con COVID-19 y evaluar las frecuencias de las variables principales.

**Materiales y métodos.:**

Se realizó un estudio de tipo observacional, transversal y analítico mediante la revisión de historias clínicas de pacientes atendidos en el Hospital Nacional Cayetano Heredia entre el año 2020 y 2021 con diagnóstico de COVID-19. Se determinó el antecedente de rinitis alérgica, y el compromiso pulmonar se evaluó mediante una tomografía sin contraste usando el puntaje tomográfico (PT), además de, variables sociodemográficas y clínicas. Se estimaron razones de prevalencias tanto crudas (RP) como ajustadas (RPa) con sus respectivos intervalos de confianza (IC) al 95% y se empleó un modelo lineal generalizado de la familia Poisson con función de enlace logarítmica y varianzas robustas.

**Resultados.:**

Se evaluaron 434 pacientes predominantemente varones, mayores de 60 años y sin antecedentes médicos relevantes. El 56,2% tenía el antecedente de rinitis alérgica y el 43,1% tuvo un compromiso pulmonar moderado a severo. En el modelo de regresión ajustado, se encontró que el antecedente de rinitis alérgica disminuyó la gravedad de COVID-19 evaluada a través del compromiso pulmonar según el PT (RPa: 0,70; IC 95%: 0,56-0,88; p=0,002).

**Conclusiones.:**

El antecedente de rinitis alérgica representó una disminución en la gravedad de COVID-19 según el PT score del 30,0% en pacientes hospitalizados.

## INTRODUCCIÓN

La enfermedad por coronavirus (COVID-19), causada por el virus SARS-CoV-2, representa un problema de salud pública que hasta diciembre del año 2022, cuenta con casi 658 millones de casos confirmados y más de seis millones de fallecidos ^1^. Debido a ello, durante la pandemia se estudiaron distintos factores de riesgo asociados a un peor pronóstico de la enfermedad, como un esfuerzo para el diseño de estrategias destinadas a una intervención temprana y evitar las hospitalizaciones y muertes. Dentro de estos factores destacan el sexo masculino, edad avanzada, tabaquismo ^2^, diabetes mellitus ^3^, hipertensión arterial (HTA), enfermedad cardiovascular, obesidad, enfermedad respiratoria crónica y cáncer ^4^. Adicionalmente a ellos, se ha sugerido que la rinitis alérgica es un factor potencial para predecir peores resultados clínicos en pacientes infectados, aunque con evidencia controversial ^5^^,^^6^. 

Una revisión sistemática mostró que los pacientes con COVID-19 y rinitis alérgica son menos propensos a la enfermedad grave y hospitalización ^7^. No obstante, en los estudios incluidos no se especificó si los pacientes tenían comorbilidades que podrían influir en la gravedad de la enfermedad y las definiciones de enfermedad grave fueron heterogéneas ^7^. Aunque las razones de esta asociación no son del todo claras, en pacientes con rinitis alérgica se encuentran concentraciones elevadas de proteína catiónica eosinófila, IL-17 e IL-33 ^8^, estando esta última presente en pacientes con cuadros severos de COVID-19 ^9^, lo que podría explicar parcialmente esta asociación. En ese sentido, considerando que esta es una enfermedad común, con una incidencia del 10 al 40% de la población mundial ^10^, su posible rol como factor de riesgo para un peor desenlace clínico de la enfermedad debería ser considerado.

Perú es uno de los países donde algunos estudios señalan que tuvo una de las mayores tasas de mortalidad por la COVID-19 a nivel mundial. Al 10 de marzo de 2023, se reportan 4 487 553 casos confirmados y 219,539 fallecidos a nivel nacional ^1^. Asimismo, en nuestro país, la prevalencia de rinitis alérgica puede llegar hasta 23% en Lima ^11^, por encima del promedio en Latinoamérica ^12^, debido a factores que incluyen una alta polución del aire ^13^. En ese sentido, identificar una posible asociación con el compromiso pulmonar de nuestros pacientes infectados podría ser relevante. A pesar de que existen estudios que intentan estimar el rol de la rinitis alérgica como posible predictor de resultados adversos en pacientes con diagnóstico de COVID-19, estos no incluyeron variables de control relevantes y se utilizaron diversos desenlaces ^7^. El puntaje tomográfico (PT) es una variable subrogada del compromiso pulmonar de los pacientes infectados y su aumento está asociado con el incremento de la mortalidad y severidad de la enfermedad ^14^. Además, predice la estancia hospitalaria y necesidad de intubación ^15^^,^^16^, por lo que consideramos que es un buen marcador para evaluar nuestra asociación. Por lo expuesto, el objetivo de nuestra investigación fue estimar la asociación entre la rinitis alérgica y el grado de compromiso pulmonar por COVID-19 en adultos atendidos en un hospital general peruano.

**MENSAJES CLAVE**

Motivación para realizar el estudio. La prevalencia de rinitis alérgica es frecuente en población peruana y se ha sugerido su asociación con la severidad de la infección por la COVID-19. Sin embargo, en estudios previos no se ha ajustado esta asociación por diversas comorbilidades, aunado al hecho de que se utilizaron diversos desenlaces.

Principales hallazgos. El antecedente de rinitis alérgica representó una disminución en la gravedad de COVID-19 según PT en pacientes hospitalizados.

Implicancias. Entre los antecedentes que se evalúan para priorizar la atención de los pacientes con COVID-19, debería incluirse la rinitis alérgica.

## MATERIALES Y MÉTODOS

### Diseño y población de estudio

El estudio es de tipo observacional, analítico y de corte transversal. Se evaluaron las historias clínicas de pacientes que se encontraban en el área de hospitalización con diagnóstico de COVID-19 atendidos en el Hospital Cayetano Heredia durante el periodo 2020-2021.

Se incluyeron las historias clínicas de pacientes adultos (18 a más años) diagnosticados con COVID-19 por evaluación clínica, prueba molecular, de antígeno o mediante una tomografía computarizada. Asimismo, definimos el antecedente de rinitis alérgica según historia clínica. Se excluyó a los pacientes cuyas historias clínicas sean ilegibles o que proporcionaron información incompleta de las variables de interés. De igual forma, se excluyó a los pacientes diagnosticados con COVID-19 por medio de una prueba rápida de anticuerpos.

De 434 historias clínicas elegibles, se obtuvieron 245 historias con diagnóstico de COVID-19 y variables de interés completas, cuyo flujograma se muestra en la Figura 1.

**Figura 1:** Flujograma de selección de la muestra analizada en el estudio.

### Cálculo del tamaño de muestra

Para el cálculo del tamaño de muestra se consideró un porcentaje de no expuestos positivos de un 43% y de expuestos positivos de 57%, con una relación entre ambos grupos de 0,74 ^6^. Se consideró un nivel de confianza de 95% y una potencia de 80%. Como resultado, se obtuvo una total de 390 historias clínicas que revisar, a las que se sumó un 10% por pérdida de información, lo que dio un total de 434 historias clínicas como muestra final, las que fueron elegidas mediante un muestreo no probabilístico por conveniencia.

### Variables

La variable independiente fue el antecedente de rinitis alérgica obtenida por medio del autorreporte del paciente. Se buscó en la historia clínica, el antecedente de rinitis alérgica obtenido por medio de la entrevista. La variable dependiente fue el grado de compromiso pulmonar por COVID-19, el que se evaluó mediante una tomografía pulmonar sin contraste tomada luego del ingreso hospitalario y se valoró por medio del PT hecha por el radiólogo que evaluó la tomografía. El PT tiene una puntuación del 0 al 25 ^16^. Los valores se categorizaron en dos grupos, de 0 a 14 y de 15 a 25, donde el primer grupo es el rango de puntaje en pacientes con compromiso leve, mientras que el segundo grupo incluyó a aquellos con compromiso moderado a severo ^15^.

Las variables sociodemográficas incluidas fueron el sexo y la edad. Asimismo, se consideraron los antecedentes médicos tales como el hábito de fumar, diabetes mellitus, hipertensión arterial, enfermedad cardiovascular ^17^, enfermedad pulmonar obstructiva crónica (EPOC), tuberculosis pulmonar e infección por el virus de la inmunodeficiencia humana (VIH). Estos antecedentes se obtuvieron directamente de la historia clínica según fueron detallados así por su médico tratante. Adicionalmente, se consideró el antecedente de alguna neoplasia y el estado de esta si la tuviera. Para evaluar el estado de la neoplasia se consideraron cuatro posibles categorías: no presenta, enfermedad neoplásica en remisión, enfermedad neoplásica activa en tratamiento y enfermedad neoplásica sin tratamiento. Además, se construyó una variable que definió la presencia de ninguna, una o dos a más comorbilidades, considerando las patologías previamente descritas (diabetes *mellitus,* HTA, enfermedad cardiovascular, EPOC, tuberculosis pulmonar e infección por VIH). Finalmente, se consideró si el paciente tenía un consumo crónico de corticoides orales o inhalados (definido como el uso por más de 10 días hasta el momento del ingreso según registro de historia clínica) ^18^.

### Análisis estadístico

Para el análisis estadístico, se utilizó la versión 16 del programa estadístico Stata (Stata Corporation, College Station, Texas, USA). Las variables categóricas fueron descritas usando frecuencias y porcentajes. Asimismo, las variables numéricas se presentaron con medidas de tendencia central y de dispersión. Para comparar las proporciones entre variables categóricas se utilizó la prueba de chi-cuadrado, así como la prueba exacta de Fisher, en caso de contar con más del 20% de valores esperados menores a 5. Además, para la comparación de medias entre dos categorías de una variable cuantitativa, se empleó la prueba t de student. Por último, se aplicó un modelo lineal generalizado de la familia Poisson con función de enlace logarítmica y varianzas robustas para estimar la asociación entre el antecedente de rinitis alérgica y el compromiso pulmonar por la COVID-19. Se elaboró un modelo ajustado basado en un criterio epidemiológico, considerando potenciales confusores descritos en la literatura. Estos confusores debían presentar asociación con la variable de exposición, desenlace y no hallarse en el camino causal. Además, se evaluó la posible colinealidad mediante un valor mayor a 10 del factor de inflación de la varianza. Se estimaron razones de prevalencias crudas (RP) y ajustadas (RPa) tomando en cuenta intervalos de confianza al 95% (IC 95%).

### Aspectos éticos

El protocolo del estudio original y el formato del cuestionario fueron aprobados por el Comité de Ética de la Universidad Peruana de Ciencias Aplicadas, el 22 de noviembre de 2021, en Lima (FCS-SCEI/1152-11-21), y por el Comité de Ética del Hospital Cayetano Heredia, el 29 de abril de 2022, en Lima (OFICIO Nº1231-2022-DG-640-OEGRRHH-360-OADI/HCH). Para la realización del presente trabajo se recolectó la información de las historias clínicas manteniendo el anonimato de los pacientes, siendo imposible así la identificación de estos en un futuro. El equipo de investigación responsable de este proyecto garantizó el respeto de los principios de autonomía, justicia y no maleficencia a lo largo de su ejecución. No existe conflicto de interés en la realización de la presente investigación y el proyecto fue registrado en la plataforma INS-PRISA (código: EI00000002619).

## RESULTADOS

Se evaluaron 434 historias clínicas. Los pacientes eran predominantemente varones, de más de 60 años sin antecedentes médicos de importancia. El 56,2% tenía el antecedente de rinitis alérgica y el 43,1% tuvo un compromiso pulmonar moderado a severo. El resto de las características generales de los datos recolectados se muestran en la Tabla 1.

**Tabla 1:** Análisis descriptivo y bivariado de las variables de interés según rinitis alérgica en pacientes con COVID-19 atendidos en el Hospital Cayetano Heredia de Lima en el periodo 2020-2021 (n=434).

Variables	N	%	Rinitis alérgica		Valor de p
			No	Sí	
			n=190 (43,8%)	n=244 (56,2%)	
Sexo					0,695 ^a^
Mujer	160	36,9	72 (45,0)	88 (55,0)	
Varón	274	63,1	118 (43,1)	67 (56,9)	
Edad					
Media ± DE	62,5 ± 13,9		66,7 ± 12,2	59,2 ± 14,3	<0,001 ^c^
18-59	161	37,1	39 (24,2)	122 (75,8)	<0,001 ^a^
60 a más	273	62,9	151 (55,3)	122 (44,7)	
Cáncer					0,978 ^b^
No presenta	408	94,0	179 (43,9)	229 (56,1)	
Enfermedad neoplásica en remisión	16	3,7	7 (43,7)	9 (56,3)	
Enfermedad neoplásica activa en tratamiento	4	0,9	2 (50,0)	2 (50,0)	
Enfermedad neoplásica activa sin tratamiento	6	1,4	2(33,3)	4(66,7)	
Fumador					0,764 ^a^
Sí	419	96,5	184 (43,9)	235 (56,1)	
No	15	3,5	6 (40,0)	9 (60,0)	
Comorbilidades					0,095 ^a^
0	178	41,0	77 (43,3)	101 (56,7)	
1	88	20,3	47 (53,4)	41 (46,6)	
2 o más	168	38,7	66 (39,3)	102 (60,7)	
Diabetes *mellitus*					0,088 ^a^
No	272	62,7	122 (44,8)	150 (55,2)	
Controlada	54	12,4	29(53,7)	25(46,3)	
No controlada	108	24,9	39(36,1)	69 (63,9)	
Enfermedad cardiovascular					0,613 ^a^
No	394	90,8	174 (44,2)	220 (55,8)	
Sí	40	9,2	16 (40,0)	24 (60,0)	
Hipertensión arterial					0,017 ^a^
No	301	69,4	133 (44,2)	168 (55,8)	
Controlada	87	20,0	45 (51,7)	42 (48,3)	
No Controlada	46	10,6	12 (26,1)	34 (73,9)	
EPOC					0,507 ^b^
No	432	99,5	190 (44,0)	242 (56,0)	
Sí	2	0,5	0 (0,0)	2 (100,0)	
Consumo crónico de corticoides					0,010 ^a^
No	343	79,1	161 (46,9)	182 (53,1)	
Sí	91	20,9	29 (31,9)	62 (68,1)	
Tuberculosis					0,475 ^b^
No	426	98,2	188 (44,1)	238 (55,9)	
Sí	8	1,8	2 (25,0)	6 (75,0)	
VIH					0,507 ^b^
No	432	99,5	190 (43,9)	242 (56,1)	
Sí	2	0,5	0 (0,0)	2 (100,0)	
Compromiso pulmonar					0,001 ^a^
Leve (0-14)	247	56,9	91 (36,8)	156 (63,2)	
Moderado/Severo (15-25)	187	43,1	99 (52,9)	88 (47,1)	

DE: desviación estándar, EPOC: enfermedad pulmonar obstructiva crónica, VIH: virus de la inmunodeficiencia humana.

a Prueba chi-Cuadrado, ^b^ prueba exacta de Fisher, ^c^ prueba t de student.

Se hallaron diferencias estadísticamente significativas en el análisis bivariado según la presencia o no de rinitis alérgica, con relación a los grupos de edad (p<0,001), el antecedente de HTA (p=0,017) y el consumo crónico de corticoides (p=0,010). En pacientes con rinitis alérgica, se halló una menor media de edad (59,2 vs. 66,7), mayor prevalencia de HTA no controlada (73,9% vs. 26,1%), de consumo crónico de corticoides (68,1% vs. 31,9%) y de compromiso pulmonar (63,2% vs. 36,8%), comparado con quienes no la presentaron (Tabla 1).

En el análisis bivariado según el compromiso pulmonar, se encontraron diferencias estadísticamente significativas en las variables edad (p=0,015) y consumo crónico de corticoides (p=0,020). En aquellos con compromiso pulmonar moderado a severo, se halló una mayor media de edad (64,4 vs. 61,1) y mayor frecuencia de consumo crónico de corticoides (53,9% vs. 46,2%) comparado con quienes tuvieron un compromiso leve (Tabla 2).

**Tabla 2:** Análisis descriptivo y bivariado de las variables de interés según compromiso pulmonar en pacientes con COVID-19 atendidos en el Hospital Cayetano Heredia de Lima en el periodo 2020-2021 (n=434).

Variables	Compromiso pulmonar		
	Leve (0-14)	Moderado/Severo (15-25)	Valor de p
	n=247 (56,9%)	n=187 (43,1%)	
Rinitis			0,001 ^a^
No	91 (47,9)	99 (52,1)	
Sí	156 (63,9)	88 (36,1)	
Sexo			0,415 ^a^
Mujer	87 (54,4)	73 (45,6)	
Varón	160 (58,4)	114 (41,6)	
Edad			
Media ± DE	61,1 ± 13,5	64,4 ± 14,3	0,015 ^c^
18-59	106 (65,8)	55 (34,2)	0,004 ^a^
60 a más	141 (51,7)	132 (48,3)	
Cáncer			0,735 ^b^
No presenta	234 (57,4)	174 (42,6)	
Enfermedad neoplásica en remisión	9 (56,3)	7 (43,7)	
Enfermedad neoplásica activa en tratamiento	2 (50,0)	2 (50,0)	
Enfermedad neoplásica activa sin tratamiento	2 (33,3)	4 (66,7)	
Fumador			0,178 ^a^
Sí	241 (57,5)	178 (42,5)	
No	6 (40,0)	9 (60,0)	
Comorbilidades			0,600 ^a^
0	106 (59,6)	72 (40,4)	
1	50 (56,8)	38 (43,2)	
2 o más	91 (54,2)	77 (45,8)	
Diabetes *mellitus*			0,974 ^a^
No	155 (57,0)	117 (43,0)	
Controlada	30 (55,6)	24 (44,4)	
No controlada	62 (57,4)	46 (42,6)	
Enfermedad cardiovascular			0,053 ^a^
No	230 (58,4)	164 (41,6)	
Sí	17 (42,5)	23 (57,5)	
Hipertensión arterial			0,916 ^a^
No	173 (57,5)	128 (42,5)	
Controlada	49 (56,3)	38 (43,7)	
No Controlada	25 (54,3)	21 (45,7)	
EPOC			0,185 ^b^
No	247 (57,1)	185 (42,9)	
Sí	0 (0,0)	2 (100,0)	
Consumo crónico de corticoides			0,020 ^a^
No	205 (59,8)	138 (40,2)	
Sí	42 (46,2)	49 (53,8)	
Tuberculosis			0,730 ^b^
No	243 (57,1)	183 (42,9)	
Sí	4 (50,0)	4 (50,0)	
VIH			0,185 ^b^
No	247 (57,1)	185 (42,9)	
Sí	0 (0,0)	2 (100,0)	

DE: desviación estándar, EPOC: enfermedad pulmonar obstructiva crónica, VIH: virus de la inmunodeficiencia humana.

a prueba chi-Cuadrado, ^b^ prueba exacta de Fisher, ^c^ prueba t de student.

**Tabla 3:** Razón de prevalencia cruda y ajustada entre rinitis alérgica y compromiso pulmonar del hospital Cayetano Heredia en Lima durante el periodo 2020-2021.

Variable	Crudo			Ajustado		
	RP	IC95%	Valor de p	RPa ^a^	IC95%	Valor de p
Rinitis alérgica						
No	Referencia			Referencia		
Sí	0,69	0,56-0,86	<0,001	0,70	0,56-0,88	0,002

RP: razón de prevalencia cruda, RPa: razón de prevalencia ajustada, IC: intervalo de confianza.

a ajustado por: sexo, edad, fumador, comorbilidades y consumo crónico de corticoides.

En el modelo crudo del análisis de regresión, se encontró que el antecedente de rinitis alérgica disminuyó la gravedad de COVID-19 (RP: 0,69; IC 95%: 0,56-0,86; p<0,001). Esta asociación mantuvo su dirección y magnitud en el modelo de regresión ajustado por sexo, edad, consumo de tabaco, comorbilidades y consumo crónico de corticoides (RPa: 0,70; IC 95%: 0,56-0,88; p=0,002) (Tabla 3). Asimismo, en el Material Suplementario se presentó el modelo de regresión crudo y ajustado completo.

## DISCUSIÓN

Nuestros principales resultados sugieren que el antecedente de rinitis alérgica se asoció a un 30,0% menor prevalencia de compromiso pulmonar moderado a severo en pacientes diagnosticados con COVID-19. 

Nuestros hallazgos son similares a una revisión sistemática de nueve estudios, que encontró que los pacientes con COVID-19 y rinitis alérgica son menos propensos a la enfermedad grave (odds ratio [OR]: 0,79, IC 95 %: 0,52-1,18; p=0,250) y hospitalización (OR: 0,23, IC 95 %: 0,02-2,67; p≤0,001) que los pacientes sin rinitis alérgica ^7^. Sin embargo, los autores señalan que los estudios analizados no incluyeron otras comorbilidades como la edad avanzada, diabetes o enfermedad cardiovascular, que, al ser factores asociados a peores resultados en estos pacientes ^19^, pueden limitar la interpretación de sus resultados. En ese sentido, el presente estudio al incluir diversas comorbilidades configura un análisis más robusto para evaluar esta asociación. Adicionalmente, a diferencia de los estudios incluidos en esa revisión sistemática, se usó el PT como desenlace clínico. Sin embargo, el PT se ha descrito como un predictor subrogado útil de mortalidad, severidad de la enfermedad ^14^, estancia hospitalaria y necesidad de intubación ^15^, por lo que es un marcador que puede ser utilizado en la práctica clínica para valorar la severidad de la enfermedad. 

Aunque, las razones por las que el antecedente de rinitis alérgica es un factor protector para el compromiso pulmonar moderado a severo en pacientes con la COVID-19 no son claras, se pueden plantear algunas hipótesis. Un estudio encontró que el alérgeno nasal del gato producía una reducción de la expresión de ARNm de la enzima convertidora de angiotensina-2 (ACE2, por sus siglas en inglés) en los cilios nasales de pacientes adultos con rinitis alérgica, que eran alérgicos a los gatos ^20^. Esto es relevante, ya que la COVID-19 utiliza un receptor ACE2 de la célula huésped para poder ingresar, la cual está presente en la mucosa oral y dentro de las vías respiratorias ^7^. Además, este receptor juega un rol notable en el desarrollo de la enfermedad, lesión pulmonar asociada y severidad de la enfermedad ^21^. De igual forma, se plantea que la exposición a IL-13 reduciría la expresión de ACE2 en células epiteliales de las vías respiratorias de personas atópicas ^22^. En este sentido, es posible que, en la rinitis alérgica también haya una disminución de la expresión de ARNm de la ACE2, lo que podría explicar esta presunta protección. Adicionalmente, es posible que no sea el antecedente de rinitis, el causante de este efecto protector, si no los medicamentos que se usan para su tratamiento como han sugerido algunos estudios. Así, algunas investigaciones sugieren una actividad antiviral directa contra el virus SARS-CoV-2 en los antagonistas de los receptores H1 al interferir con los primeros pasos de la replicación viral o al unirse a ACE2 ^23^. Por lo tanto, los pacientes que tomaban estos medicamentos tenían un riesgo significativamente menor de infección por SARS-CoV-2 ^24^. Según estudios previos, la prevalencia de rinitis alérgica en pacientes con un resultado positivo de COVID-19 es del 57,4% ^6^ que es muy similar a lo encontrado en el presente estudio.

A pesar de que los hallazgos son preliminares, éstos invitan a profundizar sobre los mecanismos, por los cuales, el antecedente de rinitis alérgica sería un posible factor protector de resultados clínicos adversos en pacientes infectados. Entender este posible mecanismo podría identificar presuntas dianas terapéuticas que permitirían crear fármacos para tratar posibles nuevas variantes de la infección u otras infecciones virales similares.

Nuestro estudio tiene como limitaciones, el usar un muestreo no probabilístico, y que los resultados no son generalizables. Al ser un estudio que toma un autorreporte, puede existir un sesgo de recuerdo, así como también, puede que al no ser una pregunta de rutina el indagar sobre el antecedente de rinitis alérgica de manera específica, existe la posibilidad de que algunos médicos indaguen este antecedente y otros no. No se incluyeron variables pertinentes como el antecedente de asma, su severidad o tratamiento en el estudio. Adicionalmente a esta limitación, el diagnóstico de rinitis alérgica no precisó el momento exacto de la enfermedad, la gravedad, su tratamiento o el tiempo desde el inicio de los síntomas hasta que se tomaron los estudios de imágenes. En casi todos los casos, las tomografías pudieron ser tomadas en momentos donde el compromiso pulmonar aún no alcanzaba su grado máximo y no se recolectó información del tiempo de hospitalización, requerimiento de unidad de cuidados intensivos (UCI), ventilación mecánica o muerte. Nuestro estudio usa el compromiso pulmonar por medio de PT como variable subrogada para evaluar la severidad del cuadro clínico por COVID-19. No obstante, los resultados plantean un potencial factor cuyos mecanismos ameritan estudiar en futuras investigaciones. 

En conclusión, más de la mitad de los pacientes tuvieron el antecedente de rinitis alérgica y cuatro de cada diez tuvo un compromiso pulmonar moderado a severo. El antecedente de rinitis alérgica tendría un efecto protector respecto al compromiso pulmonar moderado a severo de la COVID-19 y tal información serviría para realizar estudios con mayor rigurosidad científica tales como cohortes y casos y controles, además de realizar estudios de laboratorio donde se pueda encontrar el mecanismo bioquímico y molecular que explique el factor protector de la rinitis. De encontrarse el mecanismo, se podría usar con fines farmacológicos.
